# Hypertension among newly diagnosed diabetic patients at Mulago National Referral Hospital in Uganda: a cross sectional study

**DOI:** 10.5830/CVJA-2018-015

**Published:** 2018

**Authors:** Muddu Martin, Mutebi Edrisa, Ssinabulya Isaac, Kiiza Mondo Charles, Kizito Samuel

**Affiliations:** Department of Medicine, College of Health Sciences, Makerere University, Mulago Hospital Complex, Mulago, Uganda; Department of Medicine, College of Health Sciences, Makerere University, Mulago Hospital Complex, Mulago, Uganda; Department of Medicine, College of Health Sciences, Makerere University, Mulago Hospital Complex, Mulago, Uganda; Department of Medicine, College of Health Sciences, Makerere University, Mulago Hospital Complex, Mulago, Uganda; Clinical Epidemiology Unit, College of Health Sciences, Makerere University, Mulago, Uganda

**Keywords:** hypertension, newly diagnosed, diabetes, Uganda

## Abstract

**Background:**

The prevalence of hypertension in patients with diabetes is approximately two-fold higher than in age-matched subjects without the disease and, conversely, individuals with hypertension are at increased risk of developing diabetes compared with normotensive persons. Up to 75% of cases of cardiovascular disease (CVD) in patients with diabetes are attributed to hypertension. Diabetics who have hypertension are more likely to develop complications and die, and appropriate blood pressure control in these individuals reduces the risk. This study sought to determine the prevalence and factors associated with hypertension among newly diagnosed adult diabetic patients in a national referral hospital in Uganda.

**Methods:**

In this cross-sectional study, conducted between June 2014 and January 2015, we recruited 201 newly diagnosed adult diabetic patients. Information on patients’ socio-demographics was obtained using a pre-tested questionnaire, while biophysical profile, blood pressure measurement, biochemical testing and echocardiographic findings were obtained by the research team for all the participants. Bivariate and multivariate logistic regression analyses were used to investigate the association of several factors with hypertension.

**Results:**

Of the 201 patients recruited, 102 were male (50.8%) and the mean age was 46 ± 15 years. The majority of patients (159) had type 2 diabetes mellitus (DM) (79.1%) with a mean HbA_1c_ level of 13.9 ± 5.3%. The prevalence of hypertension was 61.9% (95% CI: 54.8–68.6%). Knowledge of hypertension status was at 56 (27.7%) patients, 24 (44.4%) hypertensives were on treatment, and 19 (33.9%) were using ACE inhibitors/ angiotensin receptor blockers. The independent factors associated with hypertension were being employed (OR 0.37, 95% CI: 0.16–0.90, p = 0.029) and being overweight or obese (OR 11.6, 95% CI: 4.29–31.2, p < 0.0001).

**Conclusion:**

The prevalence of hypertension was high in this population of newly diagnosed diabetics, few patients had knowledge of their hypertension status and few were on appropriate treatment. Both modifiable and non-modifiable risk factors were associated with hypertension in this group. Therefore routine assessment, treatment and control of hypertension among diabetics is necessary to prevent cardiovascular complications and death. There is also a need to address the modifiable risk factors.

The burden of non-communicable diseases (NCDs) is increasing rapidly in sub-Saharan Africa.^1^ It is anticipated that NCDs may account for 46% of deaths in sub-Saharan Africa by 2030, compared to 28% in 2008.^1^ Hypertension and diabetes mellitus (DM) are of particular concern; however, precise epidemiological data are rare.^1^-^4^ One of the commonest NCDs experienced during this early stage of the epidemiological transition is hypertension. It is predicted that more than 125 million adults in sub-Saharan Africa alone will have hypertension by 2025,^5^,^6^ and in Uganda, hypertension is the most reported NCD.^7^-^10^

Increasing urbanisation and associated lifestyle changes as well as improvements in life expectancy have contributed to a surge in NCDs, including hypertension.^1^,^5^ Likewise, the prevalence of DM is on a rise in sub-Saharan Africa and will more than double by 2025.^11^

The prevalence of hypertension in patients with diabetes is approximately two-fold higher than in age-matched subjects without the disease,^12^-^14^ and conversely, individuals with hypertension are at increased risk of developing diabetes compared with normotensive persons. Furthermore, up to 75% of cases of cardiovascular disease (CVD) in patients with diabetes can be attributed to hypertension.^15^ CVD, especially stroke, accounts for up to 80% of all deaths in the diabetic population and three-quarters of these deaths occur in sub-Saharan Africa.^16^,^17^

The high burden of hypertension in diabetics has led to an increase in the risk and prevalence of cardiac abnormalities in diabetes.^18^ Also, life expectancy in sub-Saharan Africa has risen in the past 50 years. Many more people living with diabetes are therefore exposed to the risk of hypertension for long periods for the complications to develop and for them to experience the clinical syndromes of CVD.^19^

Diabetics who have hypertension are more likely to develop complications and die, and appropriate blood pressure control in these individuals reduces the risk. The lower the systolic blood pressure, the lower the risk of complications.^12^ There is an additional risk reduction with angiotensin converting enzyme inhibitors (ACE inhibitors) and β -blockers over and above that associated with lowering of blood pressure.^12^

In patients with type 2 DM, hypertension is associated with left ventricular hypertrophy (LVH),^20^,^21^ which is an independent predictor of cardiovascular events in hypertensive patients with diabetes.^22^ Hypertension is also a major risk factor for myocardial infarction and stroke,^12^,^23^,^24^ and indeed hypertension is the leading risk factor for mortality worldwide.^5^,^25^-^28^ Additionally, hypertension is a major causal factor of end-stage kidney failure, blindness and non-traumatic amputation in people with diabetes, where attributable risks are 50, 35 and 35%, respectively.^16^

Unfortunately the majority of people with hypertension in sub-Saharan Africa do not know they have it, and most are not on treatment. This reflects the low level of knowledge of the dangers of untreated hypertension in this population.^10^

In sub-Saharan Africa there is still a lack of awareness about the growing problem of NCDs, which, unfortunately, is often coupled with the absence of a clear policy framework for prevention and management.^7^ Given the long-term decreased productivity associated with hypertension among diabetics, identifying and treating a large proportion of patients has the potential to generate tremendous social and economic benefits in this region.^5^,^29^-^31^

In this study we sought to determine the prevalence and factors associated with hypertension among newly diagnosed adult diabetic patients in a national referral hospital in Uganda. These findings are not only necessary, but also contribute to the diagnosis and management of DM and hypertension in sub-Saharan Africa.

## Methods

This study was carried out in the diabetes out-patient clinic, the medical endocrine ward and the medical emergency ward of Mulago National Referral Hospital. It is the only national referral hospital for Uganda and is the teaching hospital for Makerere University, with a bed capacity of 1 500. Mulago Hospital receives referrals from all parts of the country including from neighbouring countries such as Southern Sudan, the Democratic Republic of Congo and Rwanda. The study population is representative of the Ugandan diabetic population.

**Fig. 1:**
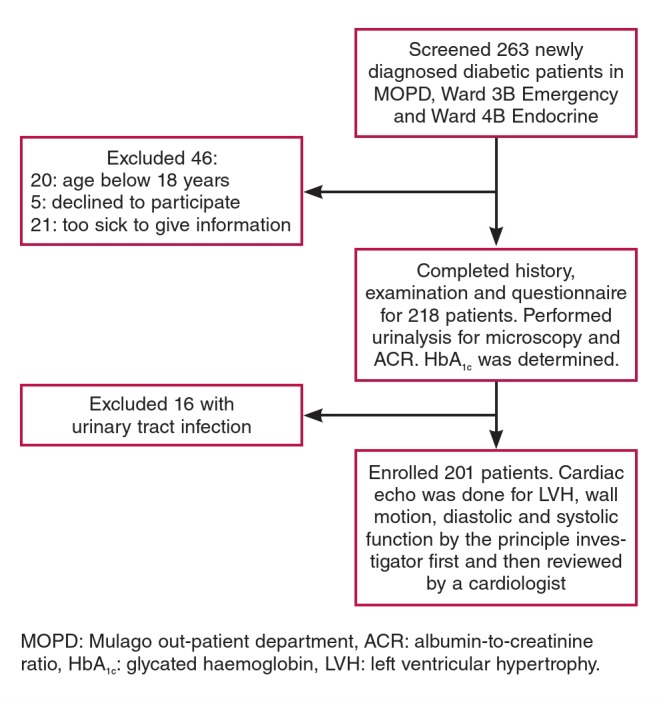
Patient flow chart.

This was a cross-sectional study among 201 newly diagnosed diabetic patients at Mulago Hospital in Uganda, conducted between June 2014 and January 2015. All newly diagnosed diabetic patients aged 18 years and above attending the diabetes clinic or admitted to the medical wards of Mulago Hospital during the study period, who met the inclusion criteria and provided informed consent, were recruited consecutively. We excluded patients with urinary tract infection in order to avoid confounding in microalbuminuria, and those who were unable to provide the necessary information. Fig. 1 illustrates the patient recruitment flow.

Institutional consent was sought from the Department of Medicine, Makerere University, Mulago National Referral Hospital and the School of Medicine research and ethics committee of Makerere University College of Health Sciences. All study participants provided written informed consent for involvement in the study. Enrolment was totally free and voluntary, and participants were free to withdraw at any time without any consequences. The patients’ records/information was anonymised and de-identified prior to analysis.

We took a focused history and performed a specific physical examination to determine biophysical measurements. Information gathered was entered into a pre-tested questionnaire. We assessed the following factors: patients’ demographic data, history of hypertension, age, physical exercise at work and leisure, marital status, date of diagnosis of DM, drug history, occupation, education level and last normal menstrual period.

Body mass was measured to the nearest kilogram using a Secco weighing scale, height was measured in metres using a non-stretchable tape, and these were used to compute body mass index (BMI). Waist and hip circumferences were measured and waist-to-hip ratios were determined for all patients.

Glycated haemoglobin (HbA_1c_) was measured by automated high-performance liquid chromatography. Other investigations included urinalysis and microalbuminuria using albumin-tocreatinine ratio.

Echocardiography parameters were acquired using a commercially available machine, Phillips HD11XE (Eindhoven, the Netherlands), with two-dimensional, M-mode and Doppler capabilities. It was used according to the American Society of Echocardiography guidelines.^32^

Blood pressure was measured using a mercury sphygmomanometer, according to the American Heart Association guidelines for the auscultatory method of blood pressure assessment.^33^ The degree of precision of blood pressure measurement in this study was ± 2 mmHg.^33^ Hypertension was defined as present if subjects were on anti-hypertensive medication, had a history of hypertension and/or evidence of hypertension (blood pressure ≥ 140/90 mmHg).

## Statistical analysis

Data were double entered in a database developed with Epidata version 3.1, validated, and inconsistences were cleared. The data were then exported to Stata 13 for analysis. Continuous data were summarised using measures of central tendency while categorical data were summarised as frequencies and percentages and presented in tables. Prevalence was presented as percentages with their confidence intervals. Comparisons were made using the Student’s t-test for continuous data and chi-squared or Fisher’s exact test for categorical data.

The outcome was dichotomised as patients having hypertension or not, then logistic regression was used to determine the association between the predictors and hypertension. This was presented as odds ratio (OR) and their 95% confidence interval (CI). Only factors with a p-value < 0.2 at bivariate analysis were considered for multivariate analysis. Multivariate logistic regression was performed and interaction was assessed for with the Chunk test. Confounding was assessed for using a 10% difference between the crude and adjusted models. Significance was at p ≤ 0.05.

## Results

This study recruited 201 newly diagnosed diabetic patients between June 2014 and January 2015. Of these, 102 (50.8%) were males. The mean age of the participants was 46 ± 15 years (Table 1). Patients with type 1 and type 2 DM had mean ages of 25.6 (18–42) and 51.9 (26–90) years, respectively. The majority of patients had type 2 DM (n = 159, 79.1%) and the rest had type 1 DM (n = 42, 20.9%) (Table 2). The mean HbA_1c_ was 13.9 ± 5.3%. Mean duration of diabetes was two months. The majority of patients (124, 62.0%) were unemployed.

**Table 1:** Social demographics of 201 newly diagnosed diabetic patients at Mulago National Referral Hospital who participated in the study

Characteristics	Total (n)	Total (%)	Hypertensive n (%)	Normotensive n (%)
Age
< 40 years	58	28.9	21 (36.2)	37 (63.8)
> 40 years	143	71.1	105 (73.4)	38 (26.6)
Gender				
Male	102	50.8	54 (52.9)	48 (47.1)
Female	99	49.3	72 (72.7)	27 (27.3)
Employment				
Employed	76	38.0	41 (53.9)	35 (46.1)
Unemployed	124	62.0	85 (68.6)	39 (31.4)
Pregnancy
Yes	6	5.4	3 (50.0)	3 (50.0)
No	105	94.6	74 (70.5)	31 (29.5)
Education				
None	17	8.5	10 (58.8)	7 (41.2)
Primary	78	38.8	50 (64.1)	28 (35.9)
Secondary	75	37.3	45 (60.0)	30 (40.0)
Tertiary	31	15.4	21 (67.7)	10 (32.3)
Marital status
Never married	29	14.4	7 (24.1)	22 (75.9)
Currently married	119	59.2	83 (69.8)	36 (30.3)
No longer married	53	26.4	36 (67.9)	17 (32.1)

**Table 2:** Characteristics of 201 newly diagnosed diabetic patients at Mulago National Referral Hospital who participated in the study

*Characteristic*	*Total (n)*	*Total (%)*	*Hypertensive n (%)*	*Normotensive n (%)*
Physical activity at work				
Sedentary	25	12.4	16 (69.6)	7 (30.4)
Mild	51	25.3	33 (64.7)	18 (35.3)
Moderate	82	40.6	54 (66.7)	27 (33.3)
Strenuous			22 (50.0)	22 (50.0)
Does not work	44	21.8	1 (50.0)	1 (50.0)
Physical activity at leisure				
Sedentary	142	71.0	96 (67.6)	46 (32.4)
Moderate	58	29.0	29 (50.0)	29 (50.0)
DM type
Type 1	42	20.9	11 (26.2)	31 (73.8)
Type 2	159	79.1	115 (72.3)	44 (27.7)
Microalbumin in urine				
Absent	79	44.9	50 (62.5)	30 (37.5)
Present	97	55.1	58 (61.1)	37 (38.3)
BMI
Underweight	39	19.4	10 (25.6)	29 (74.4)
Normal weight	75	37.3	40 (53.3)	35 (46.7)
Over weight	3	1.5	1 (33.3)	2 (66.7)
Obesity	84	41.8	75 (89.3)	9 (10.7)
Waist:hip ratio
Normal	141	69.8	81 (57.9)	59 (42.1)
Abnormal	61	30.2	45 (73.8)	16 (26.2)
HbA_1c_ (%)				
< 7%	15	8.4	11 (73.3)	4 (26.7)
> 7%	164	91.6	101 (61.9)	62 (38.0)
Ejection fraction (%)				
> 50%	158	78.2	102 (64.6)	56 (35.4)
< 50%	44	21.8	24 (55.8)	19 (44.2)
LVH				
Present	39	19.3	89 (77.4)	26 (26.5)
Absent	163	80.7	37 (43.0)	49 (56.9)
Diastolic function				
Normal	91	45.1	44 (48.9)	46 (51.1)
Impaired	111	54.9	82 (73.9)	29 (26.1)
Wall motion
Normal	193	96.5	120 (62.2)	73 (37.8)
Abnormal	7	3.5	5 (71.4)	2 (28.6)

Blood pressure assessment was performed on all 201 participants and the results are shown in Table 3. Prevalence of hypertension was 61.9% (95% CI: 54.8–68.6%). Systolic hypertension was present in 104 (51.5%) participants (95% CI: 45.3–59.2%) while diastolic hypertension was present in 92 (45.5%) (95% CI: 39.3–53.2%). Among those who were hypertensive, only 56 (27.7%) knew that they were hypertensive, and among these, only 24 (44.4%) were on treatment for hypertension. The use of either ACE inhibitors or angiotensin receptor blockers (ARBs) among those who knew their hypertension status was only 19 (33.9%) subjects.

**Table 3:** Prevalence, knowledge and treatment of hypertension among 201 newly diagnosed diabetic patients at Mulago Hospital

*Parameters*	*Number*	*Prevalence (%)*	*95% CI*
Hypertension	125	61.9	54.8–68.6
Systolic BP > 140 mmHg	104	51.5	45.3–59.2
Diastolic BP > 90 mmHg	92	45.5	39.3–53.2
Knowledge of hypertension	56	27.7	22.1–34.6
HTN newly diagnosed	69	34.2	27.6–39.8
ACEI/ARB use in known HTN	19	33.9	26.7–39.2
Known HTN on drugs	24	44.4	38.9–52.4
Known HTN not on drugs	30	55.6	47.2–62.1

HTN: hypertension, ACEI: ace inhibitor, ARB: angiotensin receptor blocker.

For participants who knew their hypertension status, the majority 44 (77.2) had been hypertensive for less than five years. The number who had been hypertensive for durations between five and 10 years and more than 10 years were eight (4.3%) and five (8.8%), respectively.

In bivariate analysis, the factors associated with hypertension included: female gender, age above 40 years, participants who were employed, participants who were never married and those who were currently married, overweight and obesity, increase in waist:hip ratio, LVH and diastolic dysfunction (Table 4). After adjusting for the patients’ gender, age, employment, marital status, BMI, waist:hip ratio, LVH and diastolic dysfunction, the only significant factors associated with hypertension were being employed (OR 0.37, 95% CI: 0.16–0.90, p = 0.029), and overweight and obesity (OR 11.6, 95% CI: 4.29–31.2, p < 0.0001).

**Table 4:** Logistic regression for factors associated with hypertension among 201 newly diagnosed diabetic patients at Mulago Hospital

	*Hypertension*					
*Factors*	*Absent, n (%)*	*Present, n (%)*	*Crude OR (95% CI)*	*p-value*	*Adjusted OR (95%)*	*p-value*
Gender
Male	48 (47.1)	54 (52.9)	1
Female	27 (27.3)	72 (72.7)	2.37 (1.32–4.27)	0.004		
Age
< 40 years	37 (63.8)	21 (36.2)	1		1	
> 40 years	38 (26.6)	105 (73.4)	4.87 (2.54–9.34)	< 0.0001	2.49 (0.62–9.95)	0.197
Employment						
Unemployed	39 (31.5)	85 (68.6)	1		1	
Employed	35 (46.1)	41 (53.9)	0.54 (0.30–0.97)	0.039	0.37 (0.16–0.90)	0.029
Marital status
No longer married	17 (32.1)	36 (67.9)	1		1	
Never married	22 (75.9)	7 (24.1)	7.25 (2.84–18.5)	< 0.0001	2.86 (0.69–11.9)	0.149
Currently married	36 (30.3)	83 (69.8)	6.66 (2.38–18.6)	< 0.0001	1.37 (0.28–6.63)	0.703
HbA_1c_
Normal	4 (26.7)	11 (73.3)	1
Abnormal	62 (38.0)	101 (61.9)	0.59 (0.18-1.94)	0.387
Microalbuminuria
Normal	30 (37.5)	50 (62.5)	1			
Abnormal	37 (38.3)	58 (61.1)	0.94 (0.51-1.74)	0.844
BMI
Normal weight	66 (56.4)	51 (43.6)	1		1	
Overweight, obesity	9 (10.7)	75 (89.3)	10.8 (4.9–23.6)	< 0.0001	11.6 (4.29–31.2)	< 0.0001
Waist:hip ratio
Normal	59 (42.1)	81 (57.9)	1		1	
Abnormal	16 (26.2)	45 (73.8)	2.05 (1.06–3.97)	0.034	1.03 (0.39–2.73)	0.949
Ejection fraction
> 50%	56 (35.4)	102 (64.6)	1
< 50%	19 (44.2)	24 (55.8)	0.69 (0.35–1.38)	0.295
LVH
Absent	49 (56.9)	37 (43.0)	1
Present	26 (26.5)	89 (77.4)	4.53 (2.46–8.35)	< 0.0001	1.97 (0.88–4.38)	0.098
Diastolic function
Normal	46 (51.1)	44 (48.9)	1		1	
Impaired	29 (26.1)	82 (73.9)	2.96 (1.64–5.34)	< 0.0001	0.94 (0.40–2.18)	0.885

HbA_1c_: glycated haemoglobin, BMI: body mass index, LVH: left ventricular hypertrophy.

## Discussion

The prevalence of hypertension among newly diagnosed diabetics was high in this group, with more than six patients out of 10 having hypertension. This is in keeping with earlier studies, which found that the prevalence of hypertension in patients with diabetes was approximately two-fold higher than in age-matched subjects without the disease,^12^-^14^ and conversely, individuals with hypertension were at increased risk of developing diabetes compared with normotensive persons.^15^

In Uganda, the prevalence of hypertension among non-diabetics ranges between 20 and 35%, with a higher prevalence in the urban areas.^10^,^34^-^37^ This is consistent with evidence from other parts of sub-Saharan Africa that indicated the prevalence of hypertension was between 20 and 50%.^7^,^35^,^37^-^40^ Therefore, the prevalence we found of 62% in diabetic subjects is approximately twice the current prevalence of hypertension in non-diabetic patients in Uganda.

Unfortunately only one-quarter of all those who are hypertensive know their status, and this is evident from other studies in the region, which found that the majority of patients with hypertension in sub-Saharan Africa did not know they were hypertensive and very few were on treatment, yet hypertension is the leading cause of stroke in Africa. In another cross-sectional study in Uganda, awareness of hypertension was low, at less than 30%.^7^

This low awareness could be explained by the fact that only 27.8% of the population ever has their blood pressure measured in Uganda. Awareness of hypertension largely depends on the capacity of the health system to provide diagnostic services for hypertension to the general population.^40^ Unfortunately, the healthcare system in Uganda is largely constrained by communicable diseases and NCDs have not received the attention they deserve.^7^ In order to increase awareness, there is a need to screen all adults at an appropriate opportunity when they come into contact with the health system. This could even be done through outreaches and community programmes.^7^,^41^,^42^

Furthermore, among those who knew they had hypertension, less than half were on treatment. This is similar to what earlier studies found, and this carries a great risk for the complications of diabetes, especially CVDs such as stroke, LVH, myocardial infarction, as reported by the United Kingdom Prospective Diabetes Study (UKPDS). In one cross-sectional study among people with hypertension in Uganda, less than 10% were controlled. In another retrospective study conducted in an urban diabetes clinic in Kampala, optimal blood pressure control, defined as ≤ 140/80 mmHg, was noted in 56% of the patients.^43^ This corroborates the notion that blood pressure control among adult diabetic patients in Uganda is sub-optimal. This calls for the development and implementation of local guidelines to improve diabetes care and minimise complications due to hypertension.^43^

Possible reasons for this very low level of control may be that the majority of people with hypertension are not aware they have the condition, and even among those who are aware, less than half are receiving treatment. However, even among those receiving treatment, only one in three achieve blood pressure control. A worrying global trend is that low levels for the control of hypertension are widespread in both low- and high-income countries.^7^,^40^,^44^,^45^

There is an additional risk reduction with ACE inhibitors and β -blockers over and above that associated with lowering of blood pressure among diabetics.^12^ However, the use of ACE inhibitors/ ARBs among those who knew their status was in only one-third of all participants, yet we know that ACE inhibitors reduce the risk for nephropathy and other complications of diabetes, such as LVH. For this reason, the JNC 7 and JNC 8 recommend that every diabetic who has hypertension must be started on ACE inhibitors/ARBs among other treatment options.^46^

In patients with type 2 DM, hypertension is associated with LVH.^20^,^21^ According to the Appropriate Blood Pressure Control in Diabetes (ABCD) trial, LVH is an independent predictor of cardiovascular events in hypertensive patients with diabetes.^22^ Hypertension is also a major risk factor for myocardial infarction and stroke,^12^,^23^,^24^ and indeed it is the leading risk factor for mortality worldwide.^5^,^25^-^27^ Therefore prevention and control of hypertension are critical in reducing morbidity and mortality attributable to cardiovascular diseases among diabetics.

According to the UKPDS, the incidence of clinical complications among diabetics is significantly associated with systolic blood pressure, except for cataract extraction. Each 10 mmHg decrease in updated mean systolic blood pressure is associated with risk reductions of 12% for any complication related to diabetes, 15% for deaths related to diabetes, 11% for myocardial infarction and 13% for microvascular complications. Any reduction in blood pressure is likely to reduce the risk of complications, with the lowest risk being in those with systolic blood pressure less than 120 mmHg.^12^

An upcoming comprehensive review of global publications on NCD costs from low- and middle-income countries confirms that primary prevention of CVD, stroke and diabetes is far less expensive and has lower unit costs than treatment interventions for these conditions. One way to achieve this is to control hypertension.^34^

The following factors were associated with hypertension among the newly diagnosed diabetics in the bivariate model: age above 40 years, female gender, unemployment, lack of physical exercise, overweight and obesity, increased waist:hip ratios, LVH and diastolic dysfunction. However after adjusting for possible confounders, only unemployment, gender and increasing BMI were independently associated with hypertension in this model. Among these factors, unemployment and BMI are modifiable, while gender is the non-modifiable factor associated with hypertension.

Attaining and maintaining a healthy weight improves blood pressure and diabetes management, and reduces cholesterol levels. The Trials of Hypertension Prevention (TOHP) study showed that a decrease of 4.4 kg can lead to a blood pressure reduction of 4/3 mmHg.^16^

In a study to determine the prevalence and factors associated with hypertension among residents of the rural district of Rukungiri, Uganda, some of the factors found to be associated with hypertension included: being overweight or obese, female gender and older age.^37^ However all these factors, apart from obesity and being overweight, had no significance in our study in the multivariate model. The reason could be that Wamala et al.^37^ in the earlier study had a bigger sample size compared to ours and enrolled community members, while our population was for newly diagnosed diabetics.

Similar findings have been reported by Wamala and co-workers^37^ and Musinguzi et al.^7^ in other cross-sectional studies. These observations suggest that demographic transition, urbanisation and increasing life expectancy are major determinants of prevalence of hypertension among diabetics.^7^,^47^-^49^

In a population-based, cross-sectional survey, Baziel et al.^1^ found further evidence to show that increasing BMI and a waist circumference above the normal range were associated with hypertension. In the same study, sociodemographic factors associated with hypertension included increasing age, male gender, overweight and obesity.

With the substantial burden of hypertension in Uganda coupled with low awareness and limited treatment of hypertension, especially among diabetics, enhanced community-based education and prevention efforts tailored to addressing modifiable factors are needed.^5^ In our study, participants who were employed were 63% less likely to have hypertension compared to their unemployed counterparts. One possible explanation would be the lack of physical exercise among the unemployed participants, whereas those who are working often do manual labour in most parts of sub-Saharan Africa.

As observed elsewhere, the prevalence of hypertension increases with increasing age, and the increase is more marked among women than men.^33^,^50^ We found age above 40 years to be associated with hypertension in the bivariate model, however this level of significance was lost in the multivariate model. With increasing life expectancy, the risk of hypertension becomes very important in sub-Saharan Africa, a region undergoing an epidemiological transition.

In addition patients who had LVH and/diastolic dysfunction were more likely to have hypertension compared to their counterparts without these heart problems. However this was no longer significant at multivariate level. One of the possible explanations could be that hypertension among diabetics caused LVH and diastolic dysfunction, as cited in the ABCD trial and other studies.^22^ Therefore treating hypertension would be one way to prevent these complications because 75% of all CVD in diabetics can be attributed to hypertension.

Microalbuminuria was not associated with hypertension in this study, despite the fact that it is one of the major CVD risk factors. Okpere et al., in a cross-sectional study among young people in the community, found contradictory evidence,^51^ but the population they studied was not diabetic.

Type 2 DM and hypertension share several common risk factors, such as physical inactivity and unhealthy diet. Overweight and obesity are potentially amenable to behavioural modification. The benefits of prevention and care extend beyond cardiovascular disease to related conditions of public health importance. They are the focus of efforts to ensure greater prioritisation of NCDs on the global research agenda as well as of development agencies and in the health and development policies of low-income countries.

## Limitations

In the diagnosis of hypertension, we did not perform ambulatory blood pressure monitoring, which is the gold standard, according to guidelines for the diagnosis of hypertension.^29^ This was due to lack of capacity. A non-diabetic control group would have provided better comparison, however in this study we assessed the prevalence and associated factors of hypertension but not its risk factors among diabetics. The recruitment time between June 2014 and January 2015 was relatively short due to limitations in logistics. This could have obscured seasonal differences.

## Conclusion

The prevalence of hypertension was high in this population of newly diagnosed diabetics, who had little knowledge of hypertension, and very few patients were on appropriate treatment. Both modifiable and non-modifiable risk factors were associated with hypertension in this group. Therefore, routine assessment, treatment and control of hypertension among diabetics is necessary to prevent CVD complications and death. Pharmacotherapy should be combined with lifestyle changes to address the modifiable risk factors.
